# Protective Effects of Ferulic Acid on Deoxynivalenol-Induced Toxicity in IPEC-J2 Cells

**DOI:** 10.3390/toxins14040275

**Published:** 2022-04-12

**Authors:** Xiangyi Meng, Wenyan Yu, Nuo Duan, Zhouping Wang, Yingbin Shen, Shijia Wu

**Affiliations:** 1 State Key Laboratory of Food Science and Technology, Jiangnan University, Wuxi 214122, China; mxy9707@126.com (X.M.); yuwenyan2018@126.com (W.Y.); duannuo@jiangnan.edu.cn (N.D.); wangzp@jiangnan.edu.cn (Z.W.); 2 School of Food Science and Technology, Jiangnan University, Wuxi 214122, China; 3 International Joint Laboratory on Food Safety, Jiangnan University, Wuxi 214122, China; 4 Innovative Center of Molecular Genetics and Evolution, School of Life Sciences, Guangzhou University, Guangzhou 510006, China; shenybin412@gmail.com

**Keywords:** ferulic acid, deoxynivalenol, oxidative stress, inflammatory, apoptosis

## Abstract

Deoxynivalenol (DON), a mycotoxin that contaminates crops such as wheat and corn, can cause severe acute or chronic injury when ingested by animals or humans. This study investigated the protective effect of ferulic acid (FA), a polyphenolic substance, on alleviating the toxicity induced by DON (40 μM) in IPEC-J2 cells. The experiments results showed that FA not only alleviated the decrease in cell viability caused by DON (*p* < 0.05), but increased the level of superoxide dismutase (SOD) (*p* < 0.01), glutathione peroxidase (GSH-Px), (catalase) CAT and glutathione (GSH) (*p* < 0.05) through the nuclear factor erythroid 2-related factor 2 (Nrf2)-epoxy chloropropane Kelch sample related protein-1 (keap1) pathway, and then decreased the levels of intracellular oxidative stress. Additionally, FA could alleviate DON-induced inflammation through mitogen-activated protein kinases (MAPKs) and nuclear factor kappa-B (NF-κB) pathways, down-regulated the secretion of interleukin-6 (IL-6) (*p* < 0.0001), interleukin-8 (IL-8) (*p* < 0.05), interleukin-1β (IL-1β), interferon-γ (IFN-γ) and further attenuated the DON-induced intracellular apoptosis (10.7% to 6.84%) by regulating the expression of Bcl2-associated X protein (Bax) (*p* < 0.0001), B-cell lymphoma-2 (Bcl-2) (*p* < 0.0001), and caspase-3 (*p* < 0.0001). All these results indicate that FA exhibits a significantly protective effect against DON-induced toxicity.

## 1. Introduction

Mycotoxins are a class of potentially toxic secondary metabolites produced by fungi. Contamination of food with mycotoxins is a serious problem facing the world, even when good storage and processing practices are adopted, which poses a major challenge to food safety [1]. DON, a Fusarium toxin, belongs to trichothecenes, and its contamination is widespread among products in different regions of the world, which seriously affects animal productivity and threatens human health [2]. Maize samples from heavy or extreme rainfall regions were observed with DON ≥ 1000 μg/kg, and wheat samples were found with DON > 1250 μg/kg in Switzerland and Luxembourg [3]. In China, wheat samples were detected with 5521.2 μg/kg, which were five-fold higher than the Chinese national standard GB 2761 [4]. DON has strong thermal stability and it is hard to destroy its composition during food processing [5]. When DON enters the human or animal body through the food chain, it will cause acute toxicity including anorexia, diarrhea, gastroenteritis, endotoxemia and even shock-like death [6]. Chronic DON intake can damage the intestinal barrier function, which can lead to nutrient absorption disorders and intestinal flora imbalance [7,8,9]. Regarding the toxicity of DON, researchers found that DON treatment decreased the antioxidative status of cells and increased the expression of genes associated with inflammation and apoptosis, such as IL-6, and IL-1β [10]. Therefore, it is necessary to find effective methods to reduce the oxidative stress, inflammation and apoptosis induced by DON to reduce its possible toxicity to humans or animals. 

Plant extracts such as dihydromyricetin, chlorogenic acid, resveratrol, and quercetin refer to a class of compounds that have a wide range of physiological activities including antioxidation, free radical scavenging and anti-UV radiation activities. In recent years, many researchers have confirmed the intervention effect of phenolic acid compounds on the toxicity of mycotoxins. Lutein and epigallocatechin-3-gallate have been proven to interfere with cell damage such as oxidative stress and inflammation caused by DON [11,12]. Resveratrol and eugenol have also been shown to improve the cytotoxicity induced by zearalenone and citrinin, respectively [13,14]. As a phenolic compound, FA is widely present in vegetables and fruits, such as tomatoes, sweet corn and rice grain [15]. It is also an effective ingredient of Chinese herbal medicine (angelica, coptis) that has the physiological effects of promoting blood circulation and removing blood stasis. Recently, a large number of studies have explored the properties, physiological functions and applications of FA and its derivatives. Bumrungpert discovered the potential of FA in reducing cardiovascular diseases [16]. Their results showed that FA reduced the oxidative stress biomarker MDA and inflammation marker tumor necrosis factor-α (TNF-α) in subjects. Another researcher explored the effect of FA on gentamicin-induced nephrotoxicity and found that FA reduced the expression of SOD, GSH, CAT, IL-6 and other stress indicators in rats, which had a protective effect on the kidneys [17]. Although some information is available on the protective role of FA in the various capacities mentioned above, the cytoprotective role against deoxynivalenol induced cytotoxicity, oxidative stress, inflammation and apoptosis has not been studied.

This study focused on how FA alleviates DON-induced cytotoxicity, oxidative stress, inflammation and apoptosis in IPEC-J2 cells, and from the related pathways, we studied the mechanism of FA through Nrf2-keap1, MAPKs, and NF-κB pathways to alleviate the intracellular state changes caused by DON, thereby determining the effectiveness of FA in reducing the damage of DON.

## 2. Results

### 2.1. FA and DON on Cell Viability of IPEC-J2 Cells

**Figure 1:**
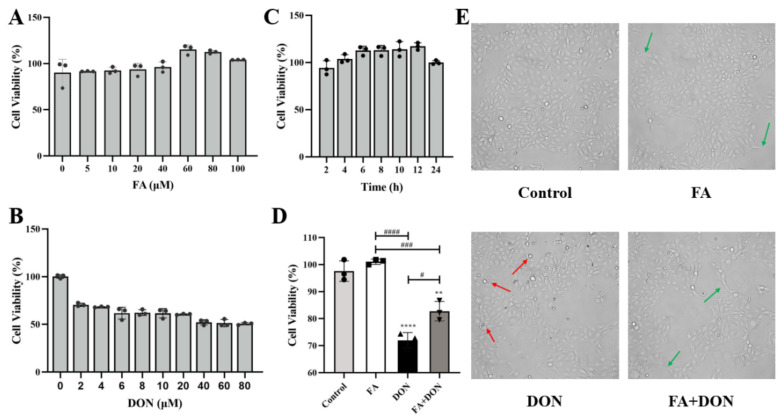
Effects of FA and DON on the viability of IPEC-J2 cells. (**A**) different concentrations of FA (**B**) different concentrations of DON (**C**) different FA processing times (**D**) FA or DON under selected experimental conditions and (**E**) cell morphology (Red arrows indicate shrunken or dead cells, green arrows indicate normal or dividing cells). The results presented are the means ± SD, ** *p* < 0.01, **** *p* < 0.0001 vs. control group, # *p* < 0.05, ### *p* < 0.001, #### *p* < 0.0001, *n* = 3. Triangles, circles and squares represent the positions of the actual values of each data point.

IPEC-J2 cells were treated with different concentrations of FA (0, 5, 10, 20, 40, 60, 80, 100 μM) or DON (0, 2, 4, 6, 8, 10, 20, 40, 60, 80 μM) alone for 12 h. The result is shown in Figure 1A,B, and FA had no obvious toxicity to IPEC-J2 cells. In results, when the FA concentration was 60 μM, the cell viability could reach a peak value of 115.16%, so 60 μM was chosen as the FA concentration for subsequent experiments. However, DON did show strong toxicity to cells. When the concentration of DON was 40 μM, cell viability reached 51.19%, close to IC_50_. Therefore, we chose 40 μM as the concentration for subsequent experiments. Then, we optimized the treatment time. As shown in Figure 1C, FA achieved a better protective effect on the cells within 12 h. Additionally, as the treatment time increased, the toxicity of DON to IPEC-J2 cells will gradually increase. Therefore, we took 12 h as the experimental time for FA and DON on IPEC-J2 cells to match the treatment time.

### 2.2. FA Alleviating DON-Induced IPEC-J2 Cell Cytotoxicity

As shown in Figure 1D, FA significantly reduced the cytotoxicity caused by DON (*p* < 0.05). Therefore, we selected the above conditions as the subsequent experimental conditions. According to the information in Figure 1E, DON exposure caused the cells to shrink or even die when suspended in the culture medium. FA pretreatment can improve this phenomenon and even enable the cells to resume division and other physiological activities.

### 2.3. FA Inhibits DON-Induced Oxidative Stress

#### 2.3.1. Elimination of ROS by FA 

**Figure 2:**
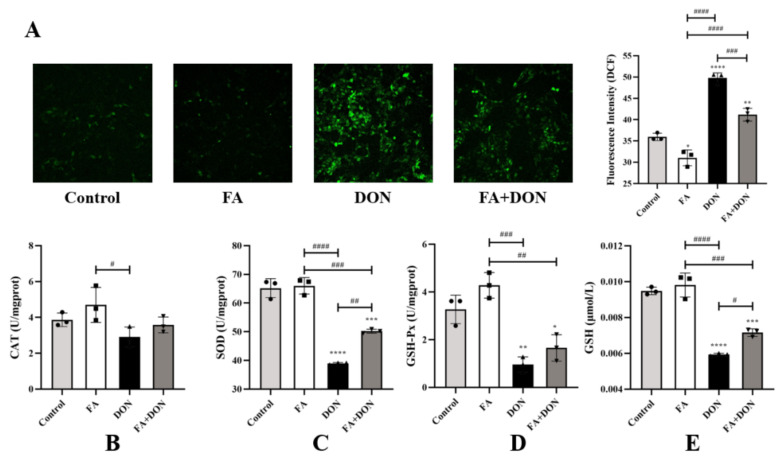
Effects of FA on DON-induced (**A**) ROS, (**B**) CAT, (**C**) SOD, (**D**) GSH-Px, and (**E**) GSH levels in IPEC-J2 cells. The results presented are the means ± SD * *p* < 0.05, ** *p* < 0.01, *** *p* < 0.001, **** *p* < 0.0001 vs. control group, # *p* < 0.05, ## *p* < 0.01, ### *p* < 0.001, #### *p* < 0.0001, *n* = 3. Triangles, circles and squares represent the positions of the actual values of each data point.

As shown in Figure 2A, compared with the control group, the FA group did not cause significant changes in intracellular ROS content. However, after DON treatment alone, the intracellular ROS content increased significantly (*p* < 0.0001). FA pretreatment effectively inhibited the generation of ROS (*p* < 0.001). This also proves that FA can inhibit DON-induced ROS production to protect cells from damage caused by free radicals.

#### 2.3.2. Effect of FA on CAT, SOD, GSH-Px, and GSH

Changes in the levels of the related antioxidant index (SOD, GSH, GSH-Px, CAT) in the cells were also detected. According to Figure 2B–E, the activity and content of the antioxidant index in FA group was roughly the same as that of the control group. However, DON exposure significantly reduced the levels of SOD (*p* < 0.0001), GSH (*p* < 0.0001), and GSH-Px (*p* < 0.01) in the IPEC-J2 cells compared with the control. Pretreatment with FA reversed this phenomenon, and the effect of SOD was the most significant (*p* < 0.01).

#### 2.3.3. Activation of the Nrf2-keap1 Signaling Pathway

**Figure 3:**
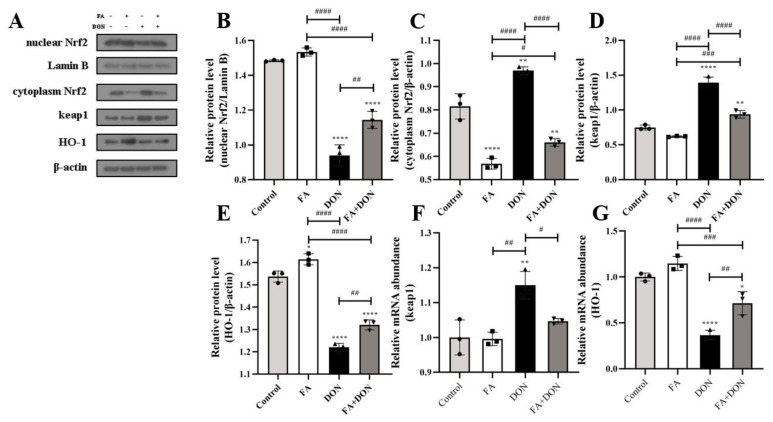
Effects of FA and DON on the activation of the Nrf2-Keap1 signaling pathways. (**A**–**E**) The expression of nuclear Nrf2, cytoplasm Nrf2, keap1, HO-1 proteins were measured by western blot. (**F**,**G**) The mRNA expression of keap1 and HO-1 were measured by RT-qPCR. The results presented are the means ± SD * *p* < 0.05, ** *p* < 0.01, **** *p* < 0.0001 vs. control group, # *p* < 0.05, ## *p* < 0.01, ### *p* < 0.001, #### *p* < 0.0001, *n* = 3. Triangles, circles and squares represent the positions of the actual values of each data point.

The effects of FA and DON on the expression of related proteins in the Nrf2-Keap1 pathway were analyzed by Western blot and quantitative real-time PCR. As shown in Figure 3A–C, when IPEC-J2 cells were exposed to DON, the Nrf2 content in the cytoplasm increased (*p* < 0.01), but the Nrf2 content in the nucleus decreased (*p* < 0.0001). This result indicates that DON inhibited the nuclear translocation of Nrf2. However, FA significantly reversed this phenomenon. After FA pretreatment, the nuclear translocation of Nrf2 is promoted (*p* < 0.01), which means that the Nrf2 pathway is activated by FA. This conclusion can also be reflected in the FA-alone treatment group. In the FA group, the Nrf2 content in the cytoplasm was significantly reduced (*p* < 0.0001). This conclusion was verified by increases in HO-1 and decreases in keap1 protein expression (*p* < 0.01, *p* < 0.0001) and mRNA abundance (*p* < 0.01, *p* < 0.05) (Figure 3F,G).

### 2.4. FA Inhibits DON-Induced Inflammation

#### 2.4.1. FA Inhibits the Production of the Inflammatory Cytokines 

**Figure 4:**
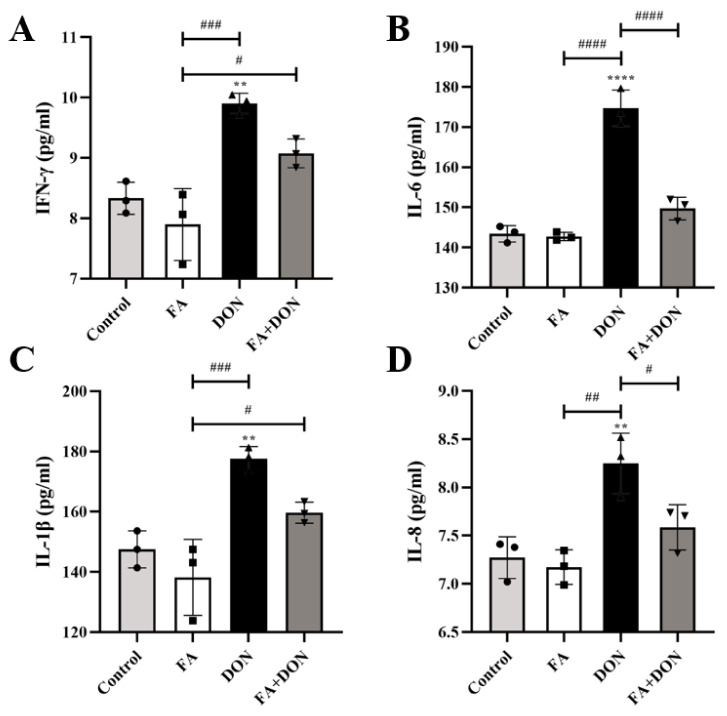
Effects of FA on DON-induced (**A**) IFN-γ, (**B**) IL-6, (**C**) IL-1β, and (**D**) IL-8 levels in IPEC-J2 cells. The results presented are the means ± SD, ** *p* < 0.01, **** *p* < 0.0001 vs. control group, # *p* < 0.05, ## *p* < 0.01, ### *p* < 0.001, #### *p* < 0.0001, *n* = 3. Triangles, circles and squares represent the positions of the actual values of each data point.

The levels of the inflammatory cytokines IFN-γ, IL-6, IL-1β, and IL-8 were measured by ELISA (Figure 4A–D). Compared with the control group, there was no significant difference in the release of each factor in the FA group. As expected, DON severely induced the inflammatory response and caused a significant increase in the production of IL-1β (*p* < 0.01), IL-6 (*p* < 0.0001), IL-8 (*p* < 0.01) and IFN-γ (*p* < 0.01) in the supernatant of IPEC-J2 culture medium. In the FA + DON group, FA pretreatment improved the above phenomenon and inhibited the release of inflammatory factors (IL-6, *p* < 0.0001, and IL-8, *p* < 0.05) caused by DON in cells.

#### 2.4.2. FA Inhibits the Phosphorylation of MAPKs Pathway Related Proteins

**Figure 5:**
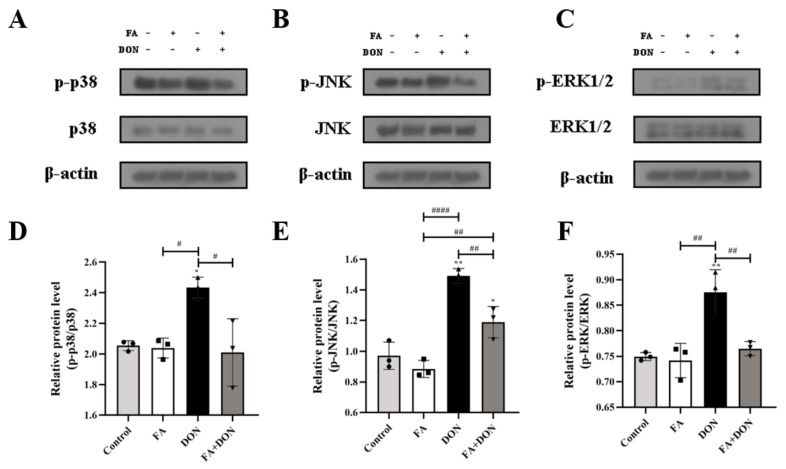
Effects of FA and DON on the activation of MAPKs signaling pathways. (**A**–**F**) The expression of p-p38, p38, p-JNK, JNK, p-ERK1/2, ERK1/2 proteins were measured by western blot. The results presented are the means ± SD * *p* < 0.05, ** *p* < 0.01, vs. control group, # *p* < 0.05, ## *p* < 0.01, #### *p* < 0.0001, *n* = 3. Triangles, circles and squares represent the positions of the actual values of each data point.

MAPKs pathway plays an important role in DON-induced inflammation in IPEC-J2 cells. Figure 5A–F displayed the changes in the expression of MAPKs pathway proteins in IPEC-J2 cells under different exposure conditions. In the DON group, the phosphorylation of p38 MAPK (*p* < 0.05), JNK (*p* < 0.01) and ERK1/2 (*p* < 0.01) was aggravated, which means that DON activated MAPKs pathway in IPEC-J2 cells. It is worth noting that in the FA + DON group, the phosphorylation level of p38 MAPK, JNK and ERK1/2 was down-regulated in the presence of FA, demonstrating that the MAPKs pathway is the target of FA to alleviate DON-induced cellular inflammation.

#### 2.4.3. FA Inhibits the Activation of the NF-κB Pathway

**Figure 6:**
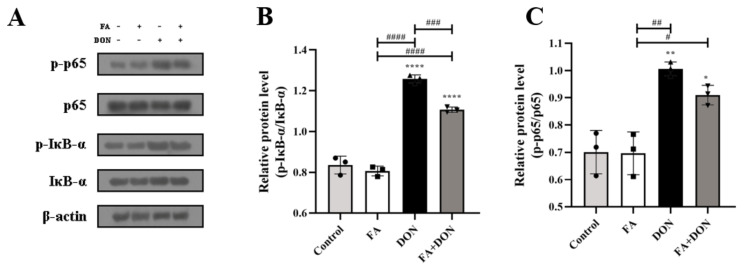
Effects of FA and DON on the activation of NF-κB signaling pathways. (**A**–**C**) The expression of p-p65, p65, p-IκB-α, IκB-α proteins were measured by western blot. The results presented are the means ± SD * *p* < 0.05, ** *p* < 0.01, **** *p* < 0.0001 vs. control group, # *p* < 0.05, ## *p* < 0.01, ### *p* < 0.001, #### *p* < 0.0001, *n* = 3. Triangles, circles and squares represent the positions of the actual values of each data point.

The activation of the NF-κB signaling pathway was investigated using Western blotting. As shown in Figure 6A–C, a stable phosphorylation of NF-κB and IκB-α FA was in FA group, whereas the phosphorylation levels were increased in DON group. These results indicated that DON had the ability to dissociate IκB-α and NF-κB, leading to the degradation of IκB-α and the transfer of NF-κB from cytoplasm into the nucleus, thus causing cellular inflammation. Fortunately, it can be found that the phosphorylation of IκB-α (*p* < 0.001) and NF-κB was suppressed in the FA treated DON group, thereby preventing the occurrence of inflammation. The results confirmed that the NF-κB pathway was also one of the action pathways for FA to interfere with DON toxicity.

### 2.5. FA Inhibits DON-Induced Apoptosis

**Figure 7:**
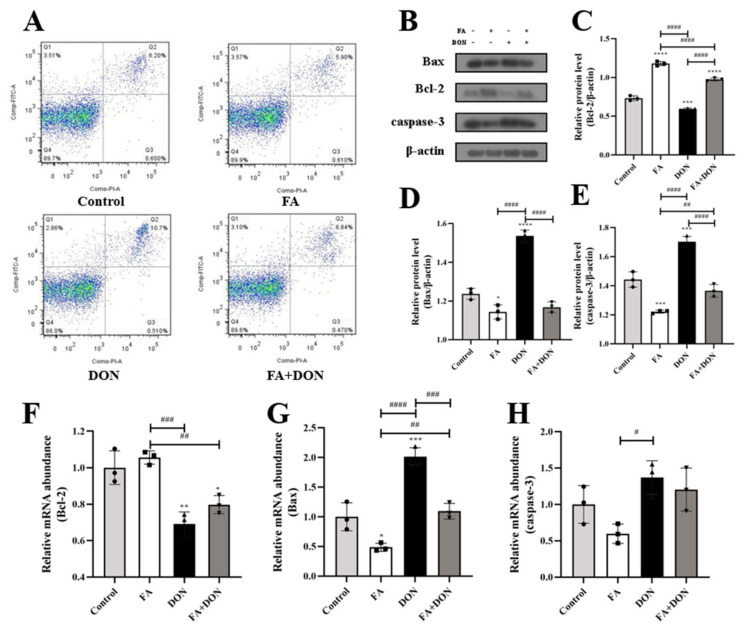
Effects of FA on DON-induced apoptosis and its pathway. (**A**) Flow cytometry analysis analysis for Annexin V/FITC/PI staining cells. (**B**–**E**) The expression of Bax, Bcl-2, caspase-3 proteins were measured by western blot. (**F**–**H**) The mRNA expression of Bax. Bcl-2, caspase-3 were measured by RT-qPCR. The results presented are the means ± SD * *p* < 0.05, ** *p* < 0.01, *** *p* < 0.001, **** *p* < 0.0001 vs. control group, # *p* < 0.05, ## *p* < 0.01, ### *p* < 0.001, #### *p* < 0.0001, *n* = 3. Triangles, circles and squares represent the positions of the actual values of each data point.

From Figure 7A, we can see that FA pretreatment can reduce the apoptosis rate from 10.7% to 6.84% compared with the DON group. On this basis, we used Western blot analysis and quantitative real-time PCR to determine the relevant indicators of the intracellular mitochondrial apoptosis pathway, as shown in Figure 7B–H. Our results confirmed that the protein content (*p* < 0.0001) and mRNA expression (*p* < 0.001) of the pro-apoptotic protein (Bax) in IPEC-J2 cells were up-regulated, whereas the protein content (*p* < 0.001) and mRNA expression (*p* < 0.01) of the anti-apoptotic protein (Bcl-2) were decreased under the condition of DON exposure alone. These results led to an increase in the protein content (*p* < 0.001) and mRNA expression of the downstream protein caspase-3, which eventually led to an increase in the apoptosis rate. After FA pretreatment, the above phenomenon was significantly reversed, and the contents (*p* < 0.0001, *p* < 0.0001, *p* < 0.0001) and mRNA expressions (*p* < 0.001 for Bax) of the above three proteins were significantly reversed.

## 3. Discussion

**Figure 8:**
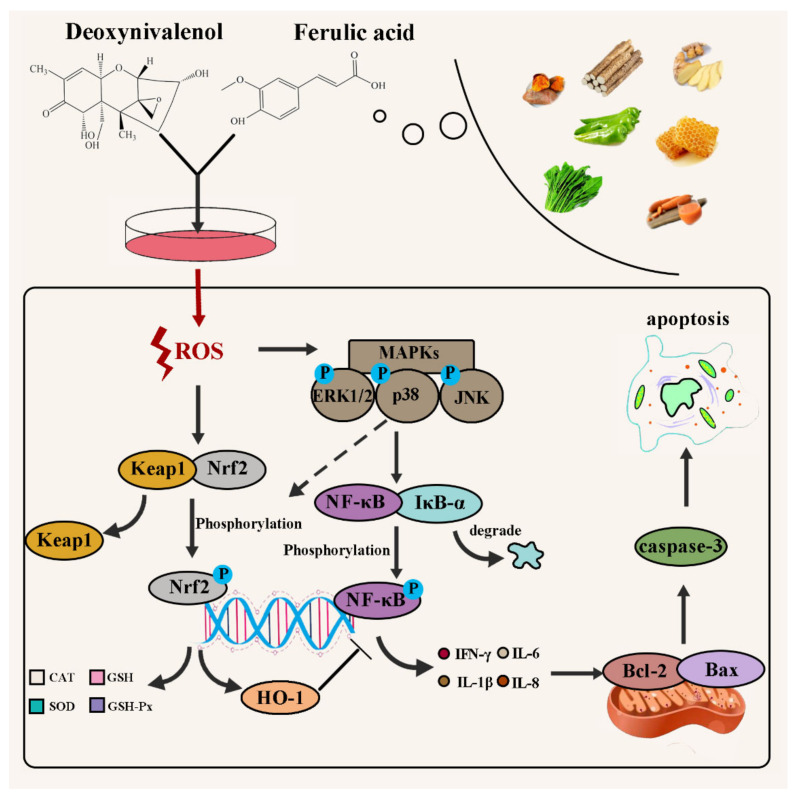
Schematic of the protective mechanism of FA on DON toxicity in vitro.

Exposure to DON can cause intracellular phospholipid peroxidation, inhibit DNA, RNA, and protein synthesis, inhibit mitochondrial function, affect cell division and the shape [18]. According to Figure 8, we can clearly understand the effect of DON on the pathway in cells. When cells were exposed to DON, it caused a significant upregulation in intracellular ROS [19]. Upregulation in ROS content can influence the Nrf2-keap1 pathway [20]. The Nrf2-keap1 pathway is the main pathway of intracellular antioxidants [21]. When the level of intracellular oxidative stress increases, Nrf2 will be activated to enter the nucleus, so that the expression of antioxidant index such as CAT, GSH, SOD, and GSH-Px will increase [22,23], thereby reducing oxidative stress. Additionally, the upregulation of ROS also increases the phosphorylation of MAPKs [24]. Subsequently, phosphorylation of the MAPKs pathway leads to activation of the NF-κB pathway [25]. That is, its inactivation after phosphorylation of its inhibitor IκB-α and nuclear translocation after phosphorylation of NF-κB [26,27]. When NF-κB is translocated to the nucleus, it will prompt cells to produce cytokines such as IL-6, IL-8, IL-1β, and IFN-γ [28,29], which will not only trigger cellular inflammation, but also further activate the mitochondrial apoptosis pathway [30,31,32], resulting in downregulation of the protein expression of antiapoptotic factor Bcl-2 and promotion of the protein expression of the apoptotic factor Bax, which further upregulates the protein expression ofcaspase-3, and finally induces cell apoptosis [19,33]. Therefore, a method that can specifically target the above DON toxicity pathway to reduce its harm to humans or animals and reduce the loss is needed.

Plant polyphenols are widespread in nature, and the unique chemical properties of their structure have fostered their biological effects such as antioxidation, anti-inflammatory effects, prevention and treatment of diabetes, anti-obesity and anti-cardio-cerebrovascular effects [34,35,36]. A large number of scholars have applied polyphenols to the detoxification of mycotoxins. Deng et al. used quercetin and tea polyphenols to feed tilapia exposed to T-2 toxin and found that the two substances reduced the liver and muscle damage in tilapia caused by T-2 toxin [37]. Ling et al. found that resveratrol could reduce the increase in cell fat and permeability by restoring the transepithelial resistance induced by DON, and enhancing the intestinal barrier function [38]. Based on the above theory, we have reason to assume that FA, which is also a phenolic compound, can also interfere with the toxicity of mycotoxins. To verify this hypothesis, by using the IPEC-J2 cell line as a model, we explored the interventional effects of FA on the intracellular toxicity of DON from three aspects: oxidative stress, inflammation and apoptosis.

In our research, the viability of IPEC-J2 cells decreased with increasing DON concentration (Figure 1B). Additionally, FA reversed the cytotoxicity induced by DON, and its activity recovered from 71.9% to 82.79%. Oxidative stress is the primary response of cells when stimulated by external factors. To further explore the intervention mechanism of FA on the intracellular oxidative stress caused by DON, we explored its effect on the Nrf2/HO-1 pathway (Figure 3). In the inactive state, Nrf2 interacts with the actin-binding protein keap1 in the cytoplasm and is rapidly degraded by the ubiquitin-proteasome pathway [39]. However, when exposed to oxidative or electrophilic stress, phosphorylation of Nrf2 leads to the dissociation of the Nrf2-keap1 complex and the stable translocation of Nrf2 to the nucleus [40]. As verified in our experiments, FA can activate the Nrf2 pathway and promote the nuclear translocation of Nrf2 to counteract the oxidative stress caused by DON exposure (Figure 3). In the nucleus, Nrf2 promotes the expression of many anti-oxidant indexes (SOD, GSH, GSH-Px, CAT) and antioxidant protein HO-1 (Figure 2 and Figure 3), which plays a vital role in cellular antioxidant responses [41,42,43,44,45,46]. Moreover, the production of HO-1 can further inhibits the nuclear translocation of NF-κB [47]. Our findings also confirm the above discussion (Figure 2 and Figure 6). In summary, FA activates the Nrf2 pathway, which phosphorylates Nrf2 and enters the nucleus from the cytoplasm, thereby causing antioxidant reactions in the cell.

Intracellular oxidative stress is closely related to the degree of inflammation [48]. Furthermore, we explored the effect of FA on the phosphorylation of the MAPKs pathway and its downstream NF-κB pathway. On the one hand, FA can inhibit the phosphorylation of p38 MAPK, ERK1/2, and JNK (Figure 5), which are subfamily of the MAPKs family [30], and un--phosphorylation p38 MAPK can promote the activation of the Nrf2-keap1 pathway to generate downstream antioxidant enzymes to alleviate the level of oxidative stress in cells [49]. On the other hand, FAcan attenuate the phosphorylation of the NF-κB inhibitor IκB-α (Figure 6), which allows unphosphorylated IκB-α to bind to NF-κB in the cytoplasm and inhibit its phosphorylation. Otherwise, NF-κB enters the nucleus, the secretion of the inflammatory factors IL-6, IL-8, IFN-γ, and IL-1β increases (Figure 4), and the level of cellular inflammation will be aggravated. The above results confirm that FA has a good intervention effect on the inflammatory reaction caused by DON.

Based on the prodsmoting relationship between MAPKs and the NF-κB pathway and apoptosis, we then explored the effect of FA on DON-induced apoptosis. Apoptosis is one of the important results of cell oxidative damage. In our research, we verified the cells treated with different conditions using flow cytometry, and the results showed that FA can significantly improve cell apoptosis caused by DON (Figure 7A). The antiapoptotic genes (Bcl-2) and proapoptotic genes (Bax) in the Bcl-2 family are also involved in the regulation of apoptosis. In our study, the expression of Bcl-2/Bax in the DON treatment group decreased significantly (Figure 7B–D) compared with that in the control group. In addition, caspase-3 is the executor of apoptosis and directly participates in apoptosis events [50]. In our study, FA had a good reversal effect on the upregulation of caspase-3 expression caused by DON (Figure 7B,E). The above results indicate that DON accelerates the process of apoptosis in cells and causes cytotoxicity. The addition of FA significantly inhibited the above trend, alleviated the tendency of cell apoptosis, and ensured cell viability.

In summary, we provided evidence that FA can attenuate DON-induced toxicity in vitro. It is verified that cytotoxicity, oxidative stress, inflammation and apoptosis are the intervention modes of FA to alleviate DON toxicity. On this basis, we clarified the intervention mechanism of FA, found Nrf-keap1, MAPKs, NF-κB and mitochondrial apoptosis pathways were its targets and analyzed the response relationship between each pathway. It was found that FA could alleviate DON-induced decrease in cell viability (71.9% to 82.7%) and changes in cell morphology. FA can also upregulate the level of intracellular antioxidant system (SOD, CAT, GSH, GSH-Px) by activating the Nrf2-keap1 pathway, and attenuate intracellular oxidative stress. In addition, we found FA can inhibit the phosphorylation of related proteins in the MAPKs pathway (p38 MAPK, JNK, ERK1/2) and the activation of the NF-κB pathway, suppressing the expression of inflammatory factors (IL-6, IL-8, IL-1β, IFN-γ) thus relieved the inflammatory response in cells. The mitochondrial apoptosis pathway in cells confirmed that FA also inhibited the abnormal expression of related apoptosis proteins, which means the mitochondrial apoptosis pathway is also an important target for FA to attenuate DON toxicity. Our study clarified the intervention effect of FA on DON-induced toxicity in vitro, and provided theoretical basis and new research direction for application of FA in the field of toxin intervention and food processing.

## 4. Materials and Methods

### 4.1. Chemicals and Reagents 

The IPEC-J2 cell line was obtained from Beina Biotechnology Co., LTD, Ferulic acid (≥99%, Aladdin,), Cell counting kit-8 (CCK-8), Annexin V-FITC/PI apoptosis detection kit was obtained from the Beyotime Biotechnology Co., Ltd. (Nantong, China). DON (purity 99%) and dimethyl sulfoxide (DMSO) were purchased from Sigma-Aldrich, Inc. (St. Louis, MO, USA). Dulbecco’s Modified Eagle’s Medium (DMEM), fetal bovine serum (FBS), antibiotics (penicillin and streptomycin), trypsin/EDTA solutions, and phosphate-buffered saline (PBS) were obtained from Gibco Laboratories (Gaithersburg, MD, USA).

### 4.2. Cell Culture and Treatments

The IPEC-J2 cells were cultured in DMEM with 10% FBS and 1% antibiotics at 37 °C under a humidified 5% CO_2_ atmosphere. When the cells confluency reached 80–90%, they were passaged for subcultures and harvest. 

In this experiment, the cells were divided into four groups: a control group, FA group, DON group and FA + DON group. The control group did not receive any treatment, the FA group and the DON group were incubated with FA (60 μM) and DON (40 μM) for 12 h, respectively, and the FA + DON group was pretreated with FA (60 μM) for 12 h before adding DON (40 μM) for 12 h. For the first 12 h, the control group and DON group were incubated with DMEM, the FA group and the FA + DON group were incubated with DMEM containing FA, and the medium of each group was discarded after the incubation. For the next 12 h, DMEM containing DON was added to the DON group and the FA + DON group to incubate the cells, and new DMEM was added to the control group and the FA group to incubate.

### 4.3. Cell Viability and Cell Morphology Observation

The cell counting kit-8 (CCK-8) was used to assess the cell viability in accordance with the manufacturer’s instructions. Briefly, IPEC-J2 cells were seeded in 96-well culture plates (5000 cells/well) and incubated for 24 h before incubation with FA or DON. Then, after replacing the original media with new media without FBS, 10 μL of CCK-8 solution was added to each well and incubated for 2 h. A microplate reader (Spectra MAX 340, 171 Molecular Devices Co., Sunnyvale, CA, USA) was used to measure the absorbance at 450 nm of every well to calculate the cell viability according to our previous method [51]. After the cells were treated with FA and DON according to the above experimental conditions, the cell morphology was observed with an inverted fluorescence microscope (Leica company, Hesse, Wetzlar, Germany).

### 4.4. Intracellular Reactive Oxygen Species (ROS) Concentration

Intracellular ROS levels were quantified by using a reactive oxygen species assay kit (Beyotime Biotechnology Co., Ltd.) according to the manufacturer’s instructions. The cells of each group were first treated differently, the cell culture was removed, and the cells were capped with 10 μM DCDF-DA that was diluted in serum-free medium. After 20 min of incubation at 37 °C, the cells were washed three times with serum-free cell culture medium to sufficiently remove DCFH-DA that did not enter the cells. Images were obtained with a confocal microscope (LSM880, Zeiss, Jena, Thuringia, Germany) and analyzed using ImageJ software. The mean fluorescence intensity was used to quantify the intracellular ROS levels. 

### 4.5. Antioxidant Index Determination

Determination of CAT, GSH-Px, and SOD activities and GSH content were carried out using kits according to the manufacturer’s instructions (Nanjing Jiancheng Bioengineering Institute, Nanjing, Jiangsu, China). The cells were cultured in 6-well plates. After treatment with FA and DON, the cells were collected and lysed on ice with an ultrasound breaker. The lysed cells were centrifuged at 10,000× *g* for 10 min at 4 °C, and the total protein concentration in the supernatant was determined using a BCA protein assay kit (Nanjing Jiancheng Bioengineering Institute). The relative activities of CAT, GSH-Px and SOD and the relative content of GSH were expressed as a ratio to total protein.

### 4.6. Enzyme-Linked Immunosorbent Assay

The cells were cultured in 6-well plates. After treatment with FA and DON, supernatants from the cell cultures were collected, and the concentrations of IL-1β, IL-6, IL-8 and INF-γ were determined using commercially available assay kits following the experimental protocols. Briefly, the supernatant was added to a microplate coated with the corresponding capture antibody, and then combined with the HRP-labeled detection antibody to form an antibody-antigen-enzyme-labeled antibody complex. After washing, TMB was added to develop color, and finally a stop solution was added. The OD value of the discolored liquid was measured at a wavelength of 450 nm and the cytokine content in the supernatant of each group was obtained according to the standard curve.

### 4.7. Flow-Cytometric Determination of Apoptosis

Flow cytometric determination of apoptosis was detected by an Annexin V-FITC-PI apoptosis detection kit (Beyotime Co., Ltd., Haimen, China). IPEC-J2 cells in each group were first treated with different conditions. Then, the treated cells were collected by centrifugation (1100 rpm, 3 min) following trypsin treatment and suspended slightly in binding buffer. Then, Annexin V-FITC and PI were added to the binding buffer-resuspended cells in the dark for 15 min. IPEC-J2 cells were analyzed by flow cytometry (BD FACSCalibur, Franklin Lake, NJ, USA). Additionally, imaging of cell apoptosis was performed with a wide field high-content analysis system (Image Xpress Micro XLS, Molecular Devices, Sunnyvale, CA, USA).

### 4.8. Quantitative Real-Time PCR

IPEC-J2 cells at a density of 5 × 10^5^ were seeded in six-well plates and incubated for 24 h to make them adherent. The processing method of each group of IPEC-J2 cells as shown in 4.2, then total RNA was extracted using a fastpure cell total RNA isolation kit (RC112-01, Vazyme, Nanjing, China). cDNA was synthesized from 1 μg of total RNA using HiScript III All-in-one RT Supermix (R333-01, Vazyme, Nanjing, China). ChamQ Universal SYBR qPCR Master Mix (Q711-02, Vazyme, Nanjing, China) (10 μL) was added to 20 μL of a reaction mixture containing cDNA (1 μL) and 0.4 μL of gene-specific forward and reverse primers (10 μM), respectively. cDNA was amplified for 40 cycles using an applied Biosystems QuantStudio 3 Flex Real-time PCR system. Glyceraldehyde-3-phosphate dehydrogenase (GAPDH) was used as a housekeeping gene for normalizing the gene levels. The expression levels of target genes were calculated using the 2^−ΔΔC^_T_ method. All the primers used in this study are listed in Table 1.

### 4.9. Western Blot Analysis

After culturing the cells in a six-well plate and mixing them with FA or DON as described above, 200 μL of 5× loading buffer was added to each well and the protein samples were collected into centrifuge tubes. The above protein samples were separated by 10–15% sodium dodecyl sulphate-polyacrylamide gel electrophoresis (SDS-PAGE). Then, the samples were transferred to a polyvinylidene difluoride membrane (PVDF). After blocking with 5% skimmed milk in Tween-20/tbs (TBST) at room temperature for 1 h, the membranes were subsequently incubated with specific primary antibodies at 4 °C overnight. The dilutions were 1:1000 (*v*/*v*) for Nrf2, keap-1, heme oxygenase-1 (HO-1), p38 MAPK, p-p38 MAPK, c-Jun N-terminal kinase (JNK), p-JNK, extracellular regulated protein kinases 1/2 (ERK1/2), P-ERK1/2, NF-κB (p65), p-NF-κB (p-p65), inhibitor of NF-κB (IκB-α), p-IκB-α, caspase-3, Bax and Bcl-2, and 1:5000 (*v*/*v*) for laminB, and β-actin. Thereafter, the membranes were probed with the HRP-conjugated secondary antibody of 1:10,000 (*v*/*v*) for 1 h at room temperature. Fluorescence was detected with an ECL Western blotting detection reagent.

### 4.10. Statistical Analysis

All experiments were repeated at least three times, and the data were analyzed by GraphPad Software (version 7.0). All the data are presented as the mean ± SD. Date were analyzed by one-way analysis of variance (ANOVA), and the Tukey test performed comparisons between groups. *p* < 0.05 is considered significant.

## Figures and Tables

**Table 1:** Primer sequences of some genes for quantitative real-time PCR.

Gene	Forward Primer (5′-3′)	Reverse Primer (5′-3′)
keap1	ATGGCGGGGCCTCTGA	CTCAGGGGCAGAAATTGGGT
HO-1	GGCTGAGAATGCCGAGTTCA	GGACGCCATCACCAGCTTAAA
Bax	GCCCTTTTGCTTCAGGGTTTC	CAATGCGCTTGAGACACTCG
Bcl-2	GATAACGGAGGCTGGGATGC	TTATGGCCCAGATAGGCACC
Caspase-3	GGAATGGCATGTCGATCTGGT	ACTGTCCGTCTCAATCCCAC
GAPDH	ATGACCACAGTCCATGCCATC	CCTGCTTCACCACCTTCTTG

## Data Availability

Not applicable.

## References

[1] Salman M.K., Mudalal S. (2022). Quality control and mycotoxin levels in food in the Palestinian market. Food Addit. Contam. Part B.

[2] Pestka J.J. (2010). Deoxynivalenol: Mechanisms of action, human exposure, and toxicological relevance. Arch. Toxicol..

[3] Gagiu V., Mateescu E., Dobre A.A., Smeu I., Cucu M.E., Oprea O.A., Alexandru D., Iorga E., Belc N. (2021). Deoxynivalenol Occurrence in Triticale Crops in Romania during the 2012–2014 Period with Extreme Weather Events. Toxins.

[4] Yan P., Liu Z., Liu S., Yao L., Liu Y., Wu Y., Gong Z. (2020). Natural Occurrence of Deoxynivalenol and Its Acetylated Derivatives in Chinese Maize and Wheat Collected in 2017. Toxins.

[5] Wu Q., Kuca K., Humpf H.U., Klimova B., Cramer B. (2017). Fate of deoxynivalenol and deoxynivalenol-3-glucoside during cereal-based thermal food processing: A review study. Mycotoxin Res..

[6] Hooft J.M., Bureau D.P. (2021). Deoxynivalenol: Mechanisms of action and its effects on various terrestrial and aquatic species. Food Chem. Toxicol..

[7] Vignal C., Djouina M., Pichavant M., Caboche S., Waxin C., Beury D., Hot D., Gower-Rousseau C., Body-Malapel M. (2018). Chronic ingestion of deoxynivalenol at human dietary levels impairs intestinal homeostasis and gut microbiota in mice. Arch. Toxicol..

[8] Qu R., Jiang C., Wu W., Pang B., Lei S., Lian Z., Shao D., Jin M., Shi J. (2019). Conversion of DON to 3-epi-DON in vitro and toxicity reduction of DON in vivo by Lactobacillus rhamnosus. Food Funct..

[9] Pinton P., Braicu C., Nougayrede J.P., Laffitte J., Taranu I., Oswald I.P. (2010). Deoxynivalenol impairs porcine intestinal barrier function and decreases the protein expression of claudin-4 through a mitogen-activated protein kinase-dependent mechanism. J. Nutr..

[10] Kang R., Li R., Dai P., Li Z., Li Y., Li C. (2019). Deoxynivalenol induced apoptosis and inflammation of IPEC-J2 cells by promoting ROS production. Environ. Pollut..

[11] Kalaiselvi P., Rajashree K., Bharathi Priya L., Padma V.V. (2013). Cytoprotective effect of epigallocatechin-3-gallate against deoxynivalenol-induced toxicity through anti-oxidative and anti-inflammatory mechanisms in HT-29 cells. Food Chem. Toxicol..

[12] Krishnaswamy R., Devaraj S.N., Padma V.V. (2010). Lutein protects HT-29 cells against Deoxynivalenol-induced oxidative stress and apoptosis: Prevention of NF-kappaB nuclear localization and down regulation of NF-kappaB and Cyclo-Oxygenase-2 expression. Free Radic. Biol. Med..

[13] Salah A., Bouaziz C., Amara I., Abid-Essefi S., Bacha H. (2019). Eugenol protects against citrinin-induced cytotoxicity and oxidative damages in cultured human colorectal HCT116 cells. Environ. Sci. Pollut. Res. Int..

[14] Sang Y., Li W., Zhang G. (2016). The protective effect of resveratrol against cytotoxicity induced by mycotoxin, zearalenone. Food Funct..

[15] Chaudhary A., Jaswal V.S., Choudhary S., Sonika, Sharma A., Beniwal V., Tuli H.S., Sharma S. (2019). Ferulic Acid: A Promising Therapeutic Phytochemical and Recent Patents Advances. Recent Pat. Inflamm. Allergy Drug Discov..

[16] Bumrungpert A., Lilitchan S., Tuntipopipat S., Tirawanchai N., Komindr S. (2018). Ferulic Acid Supplementation Improves Lipid Profiles, Oxidative Stress, and Inflammatory Status in Hyperlipidemic Subjects: A Randomized, Double-Blind, Placebo-Controlled Clinical Trial. Nutrients.

[17] Erseckin V., Mert H., Irak K., Yildirim S., Mert N. (2020). Nephroprotective effect of ferulic acid on gentamicin-induced nephrotoxicity in female rats. Drug Chem. Toxicol..

[18] Mishra S., Dixit S., Dwivedi P.D., Pandey H.P., Das M. (2014). Influence of temperature and pH on the degradation of deoxynivalenol (DON) in aqueous medium: Comparative cytotoxicity of DON and degraded product. Food Addit. Contam. Part. A Chem. Anal. Control Expo. Risk Assess..

[19] Wang X., Xu W., Fan M., Meng T., Chen X., Jiang Y., Zhu D., Hu W., Gong J., Feng S. (2016). Deoxynivalenol induces apoptosis in PC12 cells via the mitochondrial pathway. Environ. Toxicol. Pharmacol..

[20] Ji R., Jia F.Y., Chen X., Wang Z.H., Jin W.Y., Yang J. (2022). Salidroside alleviates oxidative stress and apoptosis via AMPK/Nrf2 pathway in DHT-induced human granulosa cell line KGN. Arch. Biochem. Biophys..

[21] Singh S., Vrishni S., Singh B.K., Rahman I., Kakkar P. (2010). Nrf2-ARE stress response mechanism: A control point in oxidative stress-mediated dysfunctions and chronic inflammatory diseases. Free Radic. Res..

[22] Tian Y., Li Z., Shen B., Wu L., Han L., Zhang Q., Feng H. (2017). The protective effects of Shikonin on lipopolysaccharide/d-galactosamine-induced acute liver injury via inhibiting MAPK and NF-κB and activating Nrf2/HO-1 signaling pathways. RSC Adv..

[23] Li X., Qin X., Tian J., Gao X., Wu X., Du G., Zhou Y. (2020). Liquiritin protects PC12 cells from corticosterone-induced neurotoxicity via regulation of metabolic disorders, attenuation ERK1/2-NF-kappaB pathway, activation Nrf2-Keap1 pathway, and inhibition mitochondrial apoptosis pathway. Food Chem. Toxicol..

[24] Guon T.E., Chung H.S. (2017). Moringa oleifera fruit induce apoptosis via reactive oxygen species-dependent activation of mitogen-activated protein kinases in human melanoma A2058 cells. Oncol. Lett..

[25] Zhang H., Guo Q., Liang Z., Wang M., Wang B., Sun-Waterhouse D., Waterhouse G.I.N., Wang J., Ma C., Kang W. (2021). Anti-inflammatory and antioxidant effects of Chaetoglobosin Vb in LPS-induced RAW264.7 cells: Achieved via the MAPK and NF-kappaB signaling pathways. Food Chem. Toxicol..

[26] Yuan J., Che S., Zhang L., Ruan Z. (2021). Reparative Effects of Ethanol-Induced Intestinal Barrier Injury by Flavonoid Luteolin via MAPK/NF-kappaB/MLCK and Nrf2 Signaling Pathways. J. Agric. Food Chem..

[27] Chen X., Gu M., Jin J., Ren C., Pan Z., Wu Y., Tian N., Wu A., Sun L., Gao W. (2020). beta-Hydroxyisovalerylshikonin inhibits IL-1beta-induced chondrocyte inflammation via Nrf2 and retards osteoarthritis in mice. Food Funct..

[28] Yin Z., Guo H., Jiang K., Ou J., Wang M., Huang C., Liu F., Bai W., Zheng J., Ou S. (2020). Morin decreases acrolein-induced cell injury in normal human hepatocyte cell line LO2. J. Funct. Foods.

[29] Dey D.K., Chang S.N., Kang S.C. (2021). The inflammation response and risk associated with aflatoxin B1 contamination was minimized by insect peptide CopA3 treatment and act towards the beneficial health outcomes. Environ. Pollut..

[30] Yu Y.H., Lai Y.H., Hsiao F.S., Cheng Y.H. (2021). Effects of Deoxynivalenol and Mycotoxin Adsorbent Agents on Mitogen-Activated Protein Kinase Signaling Pathways and Inflammation-Associated Gene Expression in Porcine Intestinal Epithelial Cells. Toxins.

[31] Wang X., Fan M., Chu X., Zhang Y., Rahman S.U., Jiang Y., Chen X., Zhu D., Feng S., Li Y. (2018). Deoxynivalenol induces toxicity and apoptosis in piglet hippocampal nerve cells via the MAPK signaling pathway. Toxicon.

[32] Song Q., Zhao Y., Yang Y., Han X., Duan J. (2021). Astragaloside IV protects against retinal iron overload toxicity through iron regulation and the inhibition of MAPKs and NF-kappaB activation. Toxicol. Appl. Pharmacol..

[33] Cui Y., Liu B., Sun X., Li Z., Chen Y., Guo Z., Liu H., Li D., Wang C., Zhu X. (2020). Protective effects of alfalfa saponins on oxidative stress-induced apoptotic cells. Food Funct..

[34] Xu X., Chang J., Wang P., Yin Q., Liu C., Li M., Song A., Zhu Q., Lu F. (2020). Effect of chlorogenic acid on alleviating inflammation and apoptosis of IPEC-J2 cells induced by deoxyniyalenol. Ecotoxicol. Environ. Saf..

[35] Zhong J., Yu R., Zhou Q., Liu P., Liu Z., Bian Y. (2021). Naringenin prevents TNF-alpha-induced gut-vascular barrier disruption associated with inhibiting the NF-kappaB-mediated MLCK/p-MLC and NLRP3 pathways. Food Funct..

[36] Fan J., Li B.R., Zhang Q., Zhao X.H., Wang L. (2021). Pretreatment of IEC-6 cells with quercetin and myricetin resists the indomethacin-induced barrier dysfunction via attenuating the calcium-mediated JNK/Src activation. Food Chem. Toxicol..

[37] Deng Y., Qiu M., Wang Y., Wang R., Lu P., Sun L., Li X., Gooneratne R. (2019). Protective effect of antioxidant-enriched diets on T-2-toxin-induced damage in tilapia (*Oreochromis niloticus*). Aquaculture.

[38] Yang J., Zhu C., Ye J., Lv Y., Wang L., Chen Z., Jiang Z. (2019). Protection of Porcine Intestinal-Epithelial Cells from Deoxynivalenol-Induced Damage by Resveratrol via the Nrf2 Signaling Pathway. J. Agric. Food Chem..

[39] Meng M., Zhang R., Han R., Kong Y., Wang R., Hou L. (2021). The polysaccharides from the *Grifola frondosa* fruiting body prevent lipopolysaccharide/D-galactosamine-induced acute liver injury via the miR-122-Nrf2/ARE pathways. Food Funct..

[40] Chen D., Tavana O., Gu W. (2018). ARF-NRF2: A new checkpoint for oxidative stress responses?. Mol. Cell Oncol..

[41] Zhao D., Shi D., Sun J., Li H., Zhao M., Sun B. (2018). Quantification and cytoprotection by vanillin, 4-methylguaiacol and 4-ethylguaiacol against AAPH-induced abnormal oxidative stress in HepG2 cells. RSC Adv..

[42] Long M., Yang S.H., Shi W., Li P., Guo Y., Guo J., He J.B., Zhang Y. (2017). Protective effect of proanthocyanidin on mice Sertoli cell apoptosis induced by zearalenone via the Nrf2/ARE signalling pathway. Environ. Sci. Pollut. Res. Int..

[43] Jiang Y., Zhao D., Sun J., Luo X., Li H., Sun X., Zheng F. (2019). Analysis of antioxidant effect of two tripeptides isolated from fermented grains (Jiupei) and the antioxidative interaction with 4-methylguaiacol, 4-ethylguaiacol, and vanillin. Food Sci. Nutr..

[44] Li L., Chen Y., Jiao D., Yang S., Li L., Li P. (2020). Protective Effect of Astaxanthin on Ochratoxin A-Induced Kidney Injury to Mice by Regulating Oxidative Stress-Related NRF2/KEAP1 Pathway. Molecules.

[45] Xiao Q., Piao R., Wang H., Li C., Song L. (2018). Orientin-mediated Nrf2/HO-1 signal alleviates H2O2-induced oxidative damage via induction of JNK and PI3K/AKT activation. Int. J. Biol. Macromol..

[46] Zhang J., Zhou X., Wu W., Wang J., Xie H., Wu Z. (2017). Regeneration of glutathione by alpha-lipoic acid via Nrf2/ARE signaling pathway alleviates cadmium-induced HepG2 cell toxicity. Environ. Toxicol. Pharmacol..

[47] Ahmed S.M., Luo L., Namani A., Wang X.J., Tang X. (2017). Nrf2 signaling pathway: Pivotal roles in inflammation. Biochim. Biophys. Acta Mol. Basis Dis..

[48] Jiang Y., Wang R., Yin Z., Sun J., Wang B., Zhao D., Zeng X.A., Li H., Huang M., Sun B. (2021). Optimization of Jiuzao protein hydrolysis conditions and antioxidant activity in vivo of Jiuzao tetrapeptide Asp-Arg-Glu-Leu by elevating the Nrf2/Keap1-p38/PI3K-MafK signaling pathway. Food Funct..

[49] Ko W.C., Shieh J.M., Wu W.B. (2020). P38 MAPK and Nrf2 Activation Mediated Naked Gold Nanoparticle Induced Heme Oxygenase-1 Expression in Rat Aortic Vascular Smooth Muscle Cells. Arch. Med. Res..

[50] Peng C., Sun Z., Wang L., Shu Y., He M., Ding H., Li Y., Wang X., Feng S., Li J. (2019). Soybean antigen protein induces caspase-3/mitochondrion-regulated apoptosis in IPEC-J2 cells. Food Agric. Immunol..

[51] He C., Zhou Y., Lin X., Duan N., Wang Z., Wu S. (2021). Deoxynivalenol-induced cell apoptosis monitoring using a cytochrome c-specific fluorescent probe based on a photoinduced electron transfer reaction. J. Hazard. Mater..

